# Variant and Invariant Spatiotemporal Structures in Kinematic Coordination to Regulate Speed During Walking and Running

**DOI:** 10.3389/fncom.2019.00063

**Published:** 2019-09-20

**Authors:** Hiroko Oshima, Shinya Aoi, Tetsuro Funato, Nobutaka Tsujiuchi, Kazuo Tsuchiya

**Affiliations:** ^1^Department of Mechanical and Systems Engineering, Faculty of Science and Engineering, Doshisha University, Kyoto, Japan; ^2^Department of Aeronautics and Astronautics, Graduate School of Engineering, Kyoto University, Kyoto, Japan; ^3^Department of Mechanical Engineering and Intelligent Systems, Graduate School of Informatics and Engineering, The University of Electro-Communications, Tokyo, Japan

**Keywords:** walk, run, kinematic coordination, spatiotemporal pattern, speed effect, singular value decomposition

## Abstract

Humans walk, run, and change their speed in accordance with circumstances. These gaits are rhythmic motions generated by multi-articulated movements, which have specific spatiotemporal patterns. The kinematic characteristics depend on the gait and speed. In this study, we focused on the kinematic coordination of locomotor behavior to clarify the underlying mechanism for the effect of speed on the spatiotemporal kinematic patterns for each gait. In particular, we used seven elevation angles for the whole-body motion and separated the measured data into different phases depending on the foot-contact condition, that is, single-stance phase, double-stance phase, and flight phase, which have different physical constraints during locomotion. We extracted the spatiotemporal kinematic coordination patterns with singular value decomposition and investigated the effect of speed on the coordination patterns. Our results showed that most of the whole-body motion could be explained by only two sets of temporal and spatial coordination patterns in each phase. Furthermore, the temporal coordination patterns were invariant for different speeds, while the spatial coordination patterns varied. These findings will improve our understanding of human adaptation mechanisms to tune locomotor behavior for changing speed.

## 1. Introduction

Humans walk, run, and change their speed at will depending on their circumstances. These gaits are rhythmic motions generated by multi-articulated movements that have specific spatiotemporal patterns. The kinematic characteristics of locomotor behavior vary to produce different gaits and speeds. For example, the stance leg during walking is almost straight, with slight knee flexion, and it rotates around the foot-contact point like an inverted pendulum (Lee and Farley, 1998). In contrast, the stance leg during running behaves like a spring, with knee bending (Cavagna et al., 1976). Many kinematic parameters, such as stride length and gait cycle, also change at different gaits and speeds (Nilsson et al., 1985). Such kinematic variations are locomotor outcomes of the complicated musculoskeletal system controlled by the central nervous system.

Despite large differences in locomotor behavior, there are common kinematic characteristics, which were highlighted by extracting low-dimensional structures from measured kinematics data. For example, when three elevation angles of the thigh, shank, and foot of one leg in the sagittal plane were plotted for one gait cycle, the trajectory lay close to a plane, which has been referred to as the planar law (Borghese et al., 1996; Ivanenko et al., 2007). This low-dimensional structure explains the intersegmental coordination during locomotion. In addition, the orientation of the plane constraining the trajectory changes with changes in gait and speed, suggesting that humans adapt to the speed change by tuning the intersegmental coordination (Bianchi et al., 1998; Ivanenko et al., 2007, 2008).

Human locomotion, including walking and running, is generated by moving the whole body using the legs. The legs have different roles depending on the foot-contact condition. In particular, the stance leg supports the body weight and produces propulsive and decelerative forces against the ground. In contrast, the swing leg swings the foot forward in the air and determines the stride length. Our previous work (Funato et al., 2010) investigated how spatiotemporal patterns of walking kinematics vary according to the speed by focusing on the kinematic coordination depending on the foot-contact condition. Specifically, we used seven elevation angles for the trunk and thighs, shanks, and feet of both legs, and extracted the kinematic coordination patterns using singular value decomposition for the single-stance (SS) and double-stance (DS) phases independently. As a result, a large portion of the seven angles was reproduced by the average posture and only two sets of spatial (intersegmental) and temporal coordination patterns for both phases. Furthermore, the temporal coordination patterns exhibited almost no change, while the average posture and spatial coordination patterns changed with speed.

In this study, we extended the previous analysis to running. While walking has a DS phase, running has a flight (FL) phase, in which both feet are in the air. We investigated the seven elevation angles for running for the SS and FL phases separately and examined how the kinematic coordination patterns changed with speed. We analyzed measured data for both walking and running and compared the speed effect on the spatiotemporal kinematic coordination patterns between the gaits.

## 2. Methods

### 2.1. Experiments

The study subjects were eight healthy men [age: 22–24 years, weight: 64.7 ± 6.6 kg (mean ± standard deviation), height: 1.75 ± 0.07 m]. They walked at 3, 4, and 5 km/h or ran at 9, 13, and 17 km/h on a treadmill (ITR3017, Bertec Corp.). A motion capture system (MAC3D Digital RealTime System, NAC Image Technology, Inc.) was used to measure the motion. Reflective markers were attached to the subjects' skin over several body landmarks on both the left and right sides: head, upper limit of the acromion, greater trochanter, lateral condyle of the knee, lateral malleolus, second metatarsal head, and heel. The sampling frequency was 500 Hz. This study was approved by the Ethics Committee of Doshisha University. Written informed consent was obtained from all participants after the procedures had been fully explained.

### 2.2. Analysis

**Figure 1:**
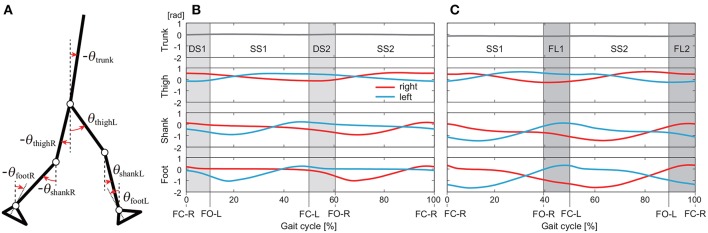
Definition of elevation angles (positive for anticlockwise direction; **A)**. Time series of elevation angles for one gait cycle composed of double-stance phases (DS1, DS2) and single-stance phases (SS1, SS2) for walking **(B)** and single-stance phases (SS1, SS2) and flight phases (FL1, FL2) for running **(C)**. These data are averages at 3 km/h for walking and 9 km/h for running by subject IG. FC-R, FC-L, FO-R, and FO-L indicate right foot contact, left foot contact, right foot off, and left foot off, respectively.

We used the measured data for 40 walking steps and 75 running steps for each subject and each speed. From the measured time-series data, we calculated the angles for seven segments (trunk and right and left feet, shanks, and thighs) defined on the sagittal plane: $\theta \left(t\right)={\left[\theta_{\text{footR}}\left(t\right)\theta_{\text{shankR}}\left(t\right)\theta_{\text{thighR}}\left(t\right)\theta_{\text{trunk}}\left(t\right)\theta_{\text{thighL}}\left(t\right)\theta_{\text{shankL}}\left(t\right)\theta_{\text{footL}}\left(t\right)\right]}^{\text{T}}\in {\mathbb{R}}^{7}$. These angles were defined as elevation angles (Figure 1A) based on the assumption that elevation angles behave more stereotypically than relative angles (Borghese et al., 1996; Ivanenko et al., 2007).

We separated the measured data into DS and SS phases for walking and FL and SS phases for running. These phases appear twice in each gait cycle, as shown in Figure 1: DS1 (starting with right foot contact), SS1 (supported on right leg), DS2 (starting with left foot contact), and SS2 (supported on left leg) for walking and SS1 (supported on right leg), FL1 (starting with right foot off), SS2 (supported on left leg), and FL2 (starting with left foot off) for running. Because SS1 and SS2, DS1 and DS2, and FL1 and FL2 are identical except for the difference in right or left, we used only DS1 and SS1 for walking and SS1 and FL1 for running. The number of data points in each phase was standardized at 100 (*t* = *t*_1_, …, *t*_100_).

We used $\Theta =\left[\theta \left(t_{1}\right)\ldots \theta \left(t_{100}\right)\right]\in {\mathbb{R}}^{7\times 100}$ for each phase by arranging the time-series data of the elevation angles θ(*t*) from *t*_1_ to *t*_100_. From singular value decomposition, we obtained

(1) $$\begin{array}{r} \Theta =\Theta_{0}+\overset{7}{\underset{i=1}{\sum }}z_{i}{\left(\lambda_{i}v_{i}\right)}^{\text{T}} \end{array}$$

where $\Theta_{0}\in {\mathbb{R}}^{7\times 100}$ was constructed by repeating the temporal average of θ(*t*), $\theta_{0}\in {\mathbb{R}}^{7}$, for 100 samplings; λ_*i*_ ∈ ℝ, $z_{i}\in {\mathbb{R}}^{7}$, and $v_{i}\in {\mathbb{R}}^{100}$ (*i* = 1, …, 7) are the singular value and the left and right singular vectors of Θ − Θ_0_, respectively; *z*_*i*_ and λ_*i*_*v*_*i*_ explain the intersegmental and temporal coordination patterns, respectively; θ_0_ is the average posture and can be decomposed into the amplitude |θ_0_| and normalized vector ${\hat{\theta }}_{0}$ (= θ_0_/|θ_0_|); and ${\hat{\theta }}_{0}$ explains the intersegmental pattern of the average posture.

To investigate the speed effect on the kinematic coordination, we used statistical methods to determine similarity for the extracted coordination patterns by singular value decomposition. To find any significant differences, we applied a multivariate analysis of variance (MANOVA) with factors speed and subject to the temporal coordination pattern λ_*i*_*v*_*i*_, intersegmental patterns *z*_*i*_ and ${\hat{\theta }}_{0}$, and normal vector of the constraint planes spanned by *z*_1_ and *z*_2_, and applied a 2-way analysis of variance (ANOVA) with factors speed and subject to the magnitude |θ_0_| of the average posture, where λ_*i*_*v*_*i*_ was converted to a vector with 25 elements. Because *z*_*i*_ and ${\hat{\theta }}_{0}$ are significant, we applied a 2-way ANOVA to each segment further, where the significance levels are based on Bonferroni correction. In addition, to compare the speed effect on the intersegmental pattern *z*_*i*_ and average posture θ_0_ between the DS and SS phases for walking and between the SS and FL phases for running, we used a paired *t*-test to the cosine similarity of the normal vectors of the constraint planes, to the cosine similarity of ${\hat{\theta }}_{0}$, and to the difference in |θ_0_| between 3 and 5 km/h for walking and between 9 and 17 km/h for running. Furthermore, to determine significant differences in the SS phase between walking and running, we applied a MANOVA with factors gait and subject to the temporal coordination patterns λ_*i*_*v*_*i*_, intersegmental coordination patterns *z*_*i*_, and average posture intersegmental pattern ${\hat{\theta }}_{0}$ and applied a 2-way ANOVA with factors gait and subject to the average posture amplitude |θ_0_|. Because *z*_*i*_ and ${\hat{\theta }}_{0}$ are significant, we also applied a 2-way ANOVA to each segment further, where the significance levels are based on Bonferroni correction. In each MANOVA and ANOVA, we confirmed that the interactions are not significant.

## 3. Results

### 3.1. Kinematic Coordination Patterns During Walking and Running

**Figure 2:**
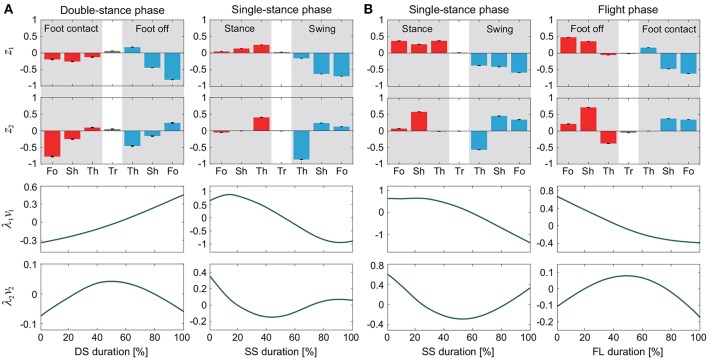
Intersegmental coordination pattern *z*_*i*_ and temporal coordination pattern λ_*i*_*v*_*i*_ obtained by singular value decomposition for walking **(A)** and running **(B)**. These data show average and standard deviation (error bar for *z*_*i*_ and gray region for λ_*i*_*v*_*i*_, too small to be visible) at 3 km/h for walking and 9 km/h for running by subject IG. Tr, Th, Sh, and Fo indicate trunk, thigh, shank, and foot, respectively.

The kinematic coordination at 3 km/h for walking and 9 km/h for running for eight subjects was analyzed using singular value decomposition. Table 1 shows the singular value λ_*i*_ and the cumulative proportion Λ_*i*_ ($=\overset{i}{\underset{j=1}{\sum }}\lambda_{j}^{2}/\overset{7}{\underset{j=1}{\sum }}\lambda_{j}^{2}$). Although the SS phase for running had a slightly smaller cumulative proportion than the other phases, most of the cumulative proportion exceeded 99% by the second coordination pattern. This indicates that the whole-body movement in each phase can be represented by only two sets of the intersegmental coordination patterns *z*_1_, *z*_2_ and temporal coordination patterns λ_1_*v*_1_, λ_2_*v*_2_ (Figure 2).

**Table 1:** Singular value λ_*i*_ and cumulative proportion Λ_*i*_ for eight subjects for each phase at 3 km/h for walking and 9 km/h for running.

**Subject**	**Walk : Double-stance phase (DS)**				**Walk : Single-stance phase (SS)**			
	**λ_1_**	**λ_2_**	**Λ_1_**	**Λ_2_**	**λ_1_**	**λ_2_**	**Λ_1_**	**Λ_2_**
IG	2.38 (0.11)	0.35 (0.04)	0.98	**1.00**	6.79 (0.17)	1.19 (0.05)	0.97	**1.00**
MT	1.84 (0.10)	0.31 (0.06)	0.97	**1.00**	6.75 (0.26)	1.51 (0.11)	0.94	**0.99**
NG	1.95 (0.10)	0.30 (0.03)	0.98	**1.00**	6.69 (0.25)	1.53 (0.09)	0.94	**0.99**
NK	1.63 (0.20)	0.24 (0.03)	0.98	**1.00**	6.96 (0.47)	1.74 (0.19)	0.93	**0.99**
ST	2.04 (0.23)	0.29 (0.05)	0.98	**1.00**	7.08 (0.41)	2.28 (0.42)	0.90	**0.99**
SG	2.21 (0.23)	0.41 (0.07)	0.96	**1.00**	6.20 (0.41)	2.03 (0.17)	0.90	**0.99**
YM	1.87 (0.09)	0.41 (0.04)	0.95	**1.00**	6.72 (0.25)	1.71 (0.07)	0.94	**1.00**
YS	2.13 (0.17)	0.46 (0.05)	0.95	**1.00**	6.29 (0.25)	1.24 (0.09)	0.96	**1.00**
**Subject**	**Run : Single-stance phase (SS)**				**Run : Flight phase (FL)**			
	**λ_1_**	**λ_2_**	**Λ_1_**	**Λ_2_**	**λ_1_**	**λ_2_**	**Λ_1_**	**Λ_2_**
IG	6.59 (0.26)	2.50 (0.17)	0.86	0.99	3.29 (0.37)	0.70 (0.08)	0.95	**1.00**
MT	6.79 (0.24)	2.47 (0.11)	0.87	0.99	2.25 (0.25)	0.46 (0.08)	0.96	**1.00**
NG	5.94 (0.27)	2.06 (0.14)	0.89	**0.99**	3.94 (0.54)	1.03 (0.16)	0.93	**0.99**
NK	5.83 (0.34)	1.86 (0.11)	0.89	0.99	3.24 (0.40)	0.45 (0.09)	0.98	**1.00**
ST	7.34 (0.39)	2.19 (0.20)	0.90	0.99	1.70 (0.29)	0.34 (0.11)	0.96	**1.00**
SG	5.86 (0.41)	2.00 (0.12)	0.89	**0.99**	3.24 (0.40)	0.77 (0.14)	0.95	**1.00**
YM	6.42 (0.29)	1.84 (0.08)	0.91	0.98	2.42 (0.25)	0.63 (0.08)	0.93	**1.00**
YS	5.66 (0.56)	2.07 (0.14)	0.86	0.98	3.31 (0.61)	0.56 (0.10)	0.97	**1.00**

*Listed values are averages and values in parentheses are standard deviations. Values are rounded off to two decimal places, and bold values denote that the cumulative proportion exceeds 99%*.

**Figure 3:**
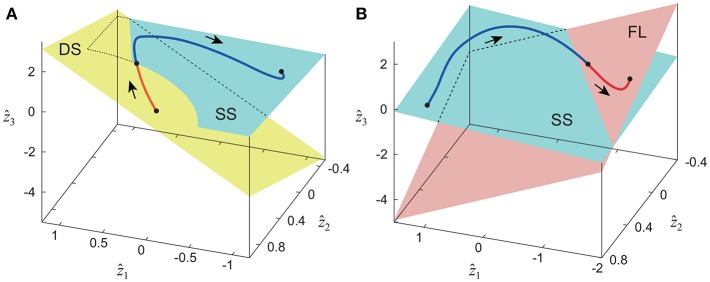
Whole-body movement and constraint planes of each phase in three-dimensional subspace for walking **(A)** and running **(B)**. Axes are given by three intersegmental coordination patterns ẑ_1_, ẑ_2_, and ẑ_3_ calculated from data of half-gait cycles DS-SS for walking and SS-FL for running. The planes are spanned by intersegmental coordination patterns *z*_1_ and *z*_2_ of each phase. These data were obtained from average at 3 km/h for walking and 9 km/h for running by subject IG. Edge points of the whole-body movement trajectory can be regarded as identical under left-right symmetry of leg movements, and the trajectory has a closed loop on these planes.

The extracted intersegmental coordination patterns *z*_1_, *z*_2_ are a subspace of the seven-dimensional space of θ(*t*). The whole-body movement lies close to the subspace in each phase and the subspace was switched between the DS and SS phases for walking and the SS and FL phases for running, depending on the foot-contact condition. To clarify this structure, we applied singular value decomposition to the data for one-half of a gait cycle by combining the DS and SS phases (DS-SS) for walking and the SS and FL phases (SS-FL) for running. Table 2 shows the singular value λ_*i*_ and the cumulative proportion Λ_*i*_. The cumulative proportion for three elements exceeded 99% in both gaits for every subject This indicates that the whole-body movement for the half-gait cycle is included in the subspace spanned by three intersegmental coordination patterns, which we call ẑ_1_, ẑ_2_, and ẑ_3_. Figure 3 shows the whole-body movement and subspaces for each phase in the three-dimensional subspaces spanned by ẑ_1_, ẑ_2_, and ẑ_3_, illustrated in the same way as in Funato et al. (2010). The coordination patterns *z*_1_, *z*_2_ span a plane for each phase in the three-dimensional subspace. The start point of the DS phase and the end point of the SS phase appear at different positions (Figure 3A). However, when the left-right symmetry of the leg movements is assumed, these two points can be regarded as identical and the whole-body movement is represented by a closed-loop trajectory on these planes. The same is true for the SS and FL phases (Figure 3B).

**Table 2:** Singular value λ_*i*_ and cumulative proportion Λ_*i*_ of eight subjects for half-gait cycle at 3 km/h for walking and 9 km/h for running.

**Subject**	**Walk : Double- and single-stance phases (DS-SS)**					

	**λ_1_**	**λ_2_**	**λ_3_**	**Λ_1_**	**Λ_2_**	**Λ_3_**
IG	6.19 (0.17)	2.96 (0.14)	0.68 (0.04)	0.80	0.99	**1.00**
MT	6.05 (0.30)	3.15 (0.17)	0.73 (0.05)	0.77	0.99	**1.00**
NG	6.01 (0.27)	3.22 (0.15)	0.72 (0.04)	0.77	0.99	**1.00**
NK	6.47 (0.45)	2.97 (0.29)	0.83 (0.09)	0.81	0.98	**1.00**
ST	6.51 (0.41)	3.71 (0.39)	0.83 (0.09)	0.74	0.99	**1.00**
SG	5.72 (0.40)	3.68 (0.22)	0.77 (0.07)	0.69	0.98	**1.00**
YM	5.97 (0.26)	3.32 (0.11)	0.69 (0.05)	0.75	0.99	**1.00**
YS	5.60 (0.25)	2.93 (0.25)	0.65 (0.06)	0.77	0.99	**1.00**
**Subject**	**Run : Single-stance and flight phases (SS-FL)**					
	**λ_1_**	**λ_2_**	**λ_3_**	**Λ_1_**	**Λ_2_**	**Λ_3_**
IG	11.01 (0.26)	2.61 (0.17)	1.31 (0.10)	0.93	0.99	**1.00**
MT	9.76 (0.22)	2.61 (0.09)	1.34 (0.07)	0.92	0.98	**1.00**
NG	10.98 (0.46)	2.40 (0.13)	1.39 (0.11)	0.94	0.98	**1.00**
NK	9.70 (0.24)	2.23 (0.11)	1.04 (0.08)	0.94	0.99	**1.00**
ST	9.75 (0.45)	2.21 (0.20)	1.33 (0.13)	0.93	0.98	**1.00**
SG	9.67 (0.25)	2.27 (0.10)	1.43 (0.12)	0.93	0.98	**1.00**
YM	9.89 (0.21)	1.91 (0.06)	1.32 (0.10)	0.95	0.98	**1.00**
YS	10.03 (0.22)	2.14 (0.11)	1.33 (0.10)	0.94	0.98	**1.00**

*Listed values are averages and values in parentheses are standard deviations. Values are rounded to two decimal places and bold values denote that the cumulative proportion exceeds 99%*.

### 3.2. Speed Effect on Kinematic Coordination

To clarify how the kinematic coordination depends on speed, we investigated the intersegmental coordination patterns *z*_1_, *z*_2_; temporal coordination patterns λ_1_*v*_1_, λ_2_*v*_2_; and average posture θ_0_ for different speeds in each phase.

**Figure 4:**
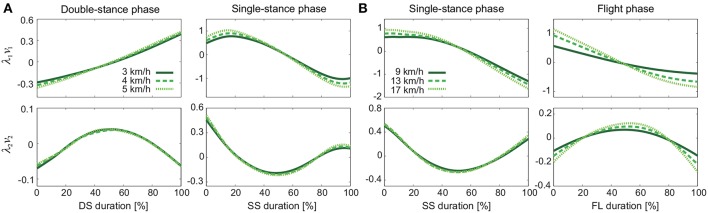
Temporal coordination pattern λ_*i*_*v*_*i*_ for each phase at 3, 4, and 5 km/h for walking **(A)** and 9, 13, and 17 km/h for running **(B)**. These are averaged data across subjects.

Figure 4 shows the temporal coordination patterns λ_1_*v*_1_, λ_2_*v*_2_ averaged across subjects for each phase at 3, 4, and 5 km/h for walking and 9, 13, and 17 km/h for running. These patterns had high similarity in each phase. To find any differences, we applied a MANOVA (Pillai's trace) with the factors of subject and speed to the discretized temporal coordination patterns, as shown in Table 3. This result shows no significant effect of speed on the temporal patterns in each phase.

**Table 3:** *P*-values of MANOVA for temporal coordination pattern λ_*i*_*v*_*i*_, MANOVA for intersegmental coordination pattern *z*_*i*_, and 2-way ANOVA for elements of *z*_*i*_.

		**Walk : DS**		**Walk : SS**	
		**1st**	**2nd**	**1st**	**2nd**
**λ_*i*_*v*_*i*_**	**MANOVA**	0.477	0.414	0.051	0.170
***z*_*i*_**	**MANOVA**	<**0.01**	<**0.01**	<**0.01**	<**0.01**
	**ANOVA**				
	Foot (R)	<**0.01/7**	<**0.01/7**	<**0.01/7**	<**0.01/7**
	Shank (R)	0.008	<**0.01/7**	<**0.01/7**	<**0.01/7**
	Thigh (R)	<**0.01/7**	<**0.01/7**	<**0.01/7**	<**0.01/7**
	Trunk	<**0.01/7**	<**0.01/7**	0.077	<**0.01/7**
	Thigh (L)	<**0.01/7**	<**0.01/7**	<**0.01/7**	<**0.01/7**
	Shank (L)	<**0.01/7**	<**0.01/7**	<**0.01/7**	<**0.01/7**
	Foot (L)	<**0.01/7**	<**0.01/7**	<**0.01/7**	<**0.01/7**
		**Run : SS**		**Run : FL**	
		**1st**	**2nd**	**1st**	**2nd**
**λ_*i*_*v*_*i*_**	**MANOVA**	0.366	0.147	0.589	0.589
***z*_*i*_**	**MANOVA**	<**0.01**	<**0.01**	<**0.01**	<**0.01**
	**ANOVA**				
	Foot (R)	<**0.01/7**	<**0.01/7**	<**0.01/7**	<**0.01/7**
	Shank (R)	<**0.01/7**	0.022	<**0.01/7**	<**0.01/7**
	Thigh (R)	<**0.01/7**	<**0.01/7**	<**0.01/7**	<**0.01/7**
	Trunk	<**0.01/7**	<**0.01/7**	<**0.01/7**	0.035
	Thigh (L)	<**0.01/7**	<**0.01/7**	<**0.01/7**	<**0.01/7**
	Shank (L)	<**0.01/7**	<**0.01/7**	<**0.01/7**	<**0.01/7**
	Foot (L)	<**0.01/7**	<**0.01/7**	<**0.01/7**	<**0.01/7**

*R and L indicate right and left, respectively. Bold values indicate significant at the 1% or 5% level*.

**Figure 5:**
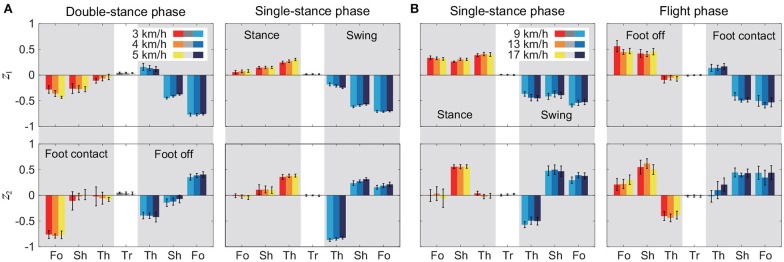
Intersegmental coordination pattern *z*_*i*_ for each phase at 3, 4, and 5 km/h for walking **(A)** and 9, 13, and 17 km/h for running **(B)**. These patterns were obtained from average and standard deviation across subjects. Tr, Th, Sh, and Fo indicate trunk, thigh, shank, and foot, respectively.

Figure 5 shows the intersegmental coordination patterns *z*_1_, *z*_2_ averaged across subjects for each phase at 3, 4, and 5 km/h for walking and 9, 13, and 17 km/h for running. These patterns also had high similarity in each phase. We applied a MANOVA (Pillai's trace) with the factors subject and speed, as shown in Table 3. Despite high similarity in appearance, this result shows that the intersegmental patterns exhibited a statistically significant effect of speed at the 1% level in each phase. Because the MANOVA was significant, we applied a 2-way ANOVA with the factors subject and speed to each segment, as shown in Table 3. The result shows that almost all speed effects are significant at 1% level in each phase and each segment. These suggest that the orientation of the constraint planes in Figure 3 changes for the speed, as obtained in our previous work for walking (Funato et al., 2010). To clarify the speed effect on the plane orientation, we calculated the normal vector of *z*_1_ and *z*_2_ in each phase in the three-dimensional subspaces spanned by ẑ_1_, ẑ_2_, and ẑ_3_ at 3 km/h for walking and 9 km/h for running in the same way in Funato et al. (2010). We applied a MANOVA (Pillai's trace) with the factors subject and speed to the normal vectors and the results showed *p* < 0.01 for all phases. In addition, we further investigated the difference of the changes in the plane orientation between the DS and SS phases for walking and between the SS and FL phases for running. Specifically, we applied a paired *t*-test to the cosine similarity of the normal vectors between 3 and 5 km/h for walking and between 9 and 17 km/h for running to determine whether the SS and DS phases have different cosine similarities for walking and whether the SS and FL phases have different cosine similarities for running, as shown in Table 4. Statistically significant differences were found at the 1% level for both walking and running. These results indicate that the constraint plane orientation changes for the speed change and that different phases have different changes in the plane orientation.

**Table 4:** Cosine similarity of normal vectors of constraint planes and *p*-values of paired *t*-test.

**Walk**			**Run**		
**DS**	**SS**	**p-value**	**SS**	**FL**	**p-value**
0.97 (0.05)	1.00 (0.00)	<**0.01**	0.99 (0.02)	0.97 (0.03)	<**0.01**

*Listed values are averages and values in parentheses are standard deviations. Bold values indicate significant at the 1% or 5% level*.

**Figure 6:**
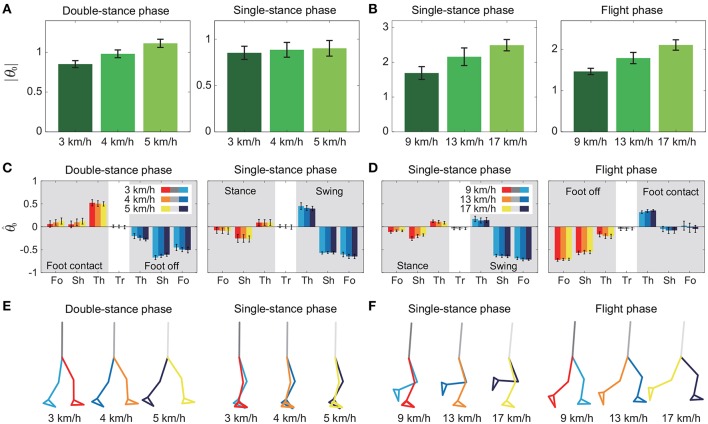
Amplitude |θ_0_| of average posture for each phase at 3, 4, and 5 km/h for walking **(A)** and 9, 13, and 17 km/h for running **(B)**. Intersegmental pattern ${\hat{\theta }}_{0}$ of average posture for each phase at 3, 4, and 5 km/h for walking **(C)** and 9, 13, and 17 km/h for running **(D)**. Tr, Th, Sh, and Fo indicate trunk, thigh, shank, and foot, respectively. Stick picture of average posture for each phase at 3, 4, and 5 km/h for walking **(E)** and 9, 13, and 17 km/h for running **(F)**. These data were obtained from average and standard deviation across subjects.

To investigate the speed effect on the average posture, we examined the amplitude |θ_0_| and intersegmental pattern ${\hat{\theta }}_{0}$. Figure 6 shows the amplitudes |θ_0_|, patterns ${\hat{\theta }}_{0}$, and stick pictures averaged across subjects for each phase at 3, 4, and 5 km/h for walking and 9, 13, and 17 km/h for running. Although the amplitudes clearly increased as the speed increased, these also showed high similarity in each phase. We applied a 2-way ANOVA with the factors subject and speed for |θ_0_| and a MANOVA (Pillai's trace) with the factors subject and speed for ${\hat{\theta }}_{0}$, as shown in Table 5. Despite high similarity in appearance, statistically significant differences were found at the 1% level in both |θ_0_| and ${\hat{\theta }}_{0}$ for all phases. Because the MANOVA was significant for ${\hat{\theta }}_{0}$, we applied a 2-way ANOVA with the factors subject and speed to each segment, as shown in Table 5. The result shows that almost all speed effects are significant at 1% level in each phase and each segment. To further clarify the speed effect on the average posture, we compared the changes in |θ_0_| and ${\hat{\theta }}_{0}$ between the DS and SS phases for walking and between the SS and FL phases for running. For |θ_0_|, we applied a paired *t*-test to the difference in |θ_0_| between 3 and 5 km/h for walking and between 9 and 17 km/h for running to determine whether the DS and SS phases have different changes for walking and whether the SS and FL phases have different changes for running, as shown in Table 6. Statistically significant differences were found at the 1% level for both walking and running. For ${\hat{\theta }}_{0}$, we applied a paired *t*-test to the cosine similarity of ${\hat{\theta }}_{0}$ at 3 and 5 km/h for walking and 9 and 17 km/h for running to determine whether the DS and SS phases have different cosine similarities for walking and the SS and FL phases have different cosine similarities for running, as shown in Table 6. Statistically significant differences were found at the 1% level for both walking and running. These results indicate that both the amplitude and intersegmental pattern of the average posture change for the speed changes and that different phases have different changes in the average posture characteristics.

**Table 5:** *P*-values of 2-way ANOVA for average posture amplitude |θ_0_|, MANOVA for average posture intersegmental pattern ${\hat{\theta }}_{0}$, and 2-way ANOVA for elements of ${\hat{\theta }}_{0}$.

		**Walk**		**Run**	
		**DS**	**SS**	**SS**	**FL**
**|θ_0_|**	**ANOVA**	<**0.01**	<**0.01**	<**0.01**	<**0.01**
${\hat{\theta }}_{0}$	**MANOVA**	<**0.01**	<**0.01**	<**0.01**	<**0.01**
	**ANOVA**				
	Foot (R)	<**0.01/7**	<**0.01/7**	<**0.01/7**	<**0.01/7**
	Shank (R)	<**0.01/7**	<**0.01/7**	<**0.01/7**	<**0.01/7**
	Thigh (R)	<**0.01/7**	0.928	<**0.01/7**	<**0.01/7**
	Trunk	<**0.01/7**	<**0.01/7**	<**0.01/7**	<**0.01/7**
	Thigh (L)	<**0.01/7**	<**0.01/7**	<**0.01/7**	<**0.01/7**
	Shank (L)	<**0.01/7**	<**0.05/7**	<**0.01/7**	<**0.01/7**
	Foot (L)	<**0.01/7**	<**0.01/7**	<**0.01/7**	<**0.01/7**

*R and L indicate right and left, respectively. Bold values indicate significant at the 1% or 5% level*.

**Table 6:** Difference of average posture amplitude |θ_0_|, cosine similarity of average posture intersegmental pattern ${\hat{\theta }}_{0}$, and *p*-values of paired *t*-test.

	**Walk**			**Run**		
	**DS**	**SS**	**p-value**	**SS**	**FL**	**p-value**
**|θ_0_|**	0.25 (0.06)	0.05 (0.08)	<**0.01**	0.77 (0.23)	0.63 (0.10)	<**0.01**
${\hat{\theta }}_{0}$	0.98 (0.01)	0.99 (0.01)	<**0.01**	0.99 (0.01)	0.99 (0.01)	<**0.01**

*Listed values are averages and values in parentheses are standard deviations. Bold values indicate significant at the 1% or 5% level*.

### 3.3. Comparison Between Walking and Running

Because both walking and running have the SS phase, we compared the kinematic coordination between 3 km/h for walking and 9 km/h for running. We applied a MANOVA (Pillai's trace) for the temporal coordination patterns λ_1_*v*_1_, λ_2_*v*_2_; intersegmental coordination patterns *z*_1_, *z*_2_; and average posture intersegmental pattern ${\hat{\theta }}_{0}$ and applied a paired *t*-test for the average posture amplitude |θ_0_|, as shown in Table 7. Although the temporal coordination patterns had no apparent difference between the gaits, the others had statistically significant differences at the 1% level. Because the MANOVA was significant for ${\hat{\theta }}_{0}$, we applied a 2-way ANOVA with the factors subject and gait to each segment, as shown in Table 7. The result shows that almost all gait effects are significant at 1% level in each phase and each segment.

**Table 7:** *P*-values of MANOVA for temporal coordination pattern λ_*i*_*v*_*i*_, intersegmental coordination pattern *z*_*i*_, and average posture intersegmental pattern ${\hat{\theta }}_{0}$ and 2-way ANOVA for elements of *z*_*i*_, ${\hat{\theta }}_{0}$, and average posture amplitude |θ_0_| to determine significant differences in the SS phase between walking and running.

	****|θ_0_|****				
**ANOVA**	<**0.01**				
	**λ_1_*v*_1_**	**λ_2_*v*_2_**	***z*_1_**	***z*_2_**	${\hat{\theta }}_{0}$
**MANOVA**	0.166	0.114	<**0.01**	<**0.01**	<**0.01**
**ANOVA**					
Foot (R)	–	–	<**0.01/7**	<**0.05/7**	<**0.01/7**
Shank (R)	–	–	<**0.01/7**	<**0.01/7**	0.163
Thigh (R)	–	–	<**0.01/7**	<**0.01/7**	<**0.01/7**
Trunk	–	–	<**0.01/7**	<**0.01/7**	<**0.01/7**
Thigh (L)	–	–	<**0.01/7**	<**0.01/7**	<**0.01/7**
Shank (L)	–	–	<**0.01/7**	<**0.01/7**	<**0.01/7**
Foot (L)	–	–	<**0.01/7**	<**0.01/7**	<**0.01/7**

*Bold values indicate significant at the 1% or 5% level*.

## 4. Discussion

In this study, we analyzed the whole-body movement using measurements taken during walking and running. In particular, the time series of seven elevation angles were decomposed by singular value decomposition after being separated into the DS and SS phases for walking and SS and FL phases for running (Figure 1). The whole-body movement was revealed to be composed of the average posture and only two sets of principal intersegmental and temporal coordination patterns irrespective of the phase and gait (Table 1). We investigated the relationship between the coordination patterns and gait speed to clarify the underlying mechanism for adapting the whole-body movement to the speed change at each phase.

Previous works (Borghese et al., 1996; Ivanenko et al., 2008) have shown that three elevation angles for one leg in one gait cycle can be described by a closed loop on a plane. Such planar covariation of the elevation angles held for various gaits, such as running (Hicheur et al., 2006; Ivanenko et al., 2007), curved walking (Courtine and Schieppati, 2004), backward walking (Grasso et al., 1998; Hicheur et al., 2006), walking on inclined surfaces (Noble and Prentice, 2008), walking with bent or erect posture (Grasso et al., 2000), stepping over an obstacle (Ivanenko et al., 2005b; Maclellan and McFadyen, 2010), walking with body weight unloading (Ivanenko et al., 2002), and walking on a slippery surface (Cappellini et al., 2010), which suggests an invariant characteristic in locomotion. In addition, this characteristic appeared in toddlers (Cheron et al., 2001; Ivanenko et al., 2004, 2005a; Dominici et al., 2007, 2010; Hallemans and Aerts, 2009; Cappellini et al., 2016); neonates (Dominici et al., 2011); gait disorders (Grasso et al., 2004; Laroche et al., 2007; Leurs et al., 2012; Martino et al., 2014; Cappellini et al., 2016; Ishikawa et al., 2017; Wallard et al., 2018); and also various animals (Catavitello et al., 2018), including cats (Poppele and Bosco, 2003), dogs (Catavitello et al., 2015), monkeys (Courtine et al., 2005; Ogihara et al., 2012), and birds (Ogihara et al., 2014). Investigating the coordination structures has provided useful insights for adaptation mechanisms in locomotion. To reveal a more detailed structure of kinematic coordination during human walking and running, we used seven angles of the whole body, including the trunk, and separated the measured data depending on the foot-contact condition. Because relative joint angles are not stereotypical across subjects and are more variable than elevation angles, the planarity of the joint angles is weaker for the analysis of three angles of one leg (Borghese et al., 1996; Ivanenko et al., 2007). Extracting the low-dimensional structure depends on the coordinate system (Yamasaki et al., 2013). We used the elevation angles for the analysis and our results showed that most of the cumulative proportion exceeded 99% by the second coordination pattern (Table 1), which indicates that we successfully extracted the low-dimensional structure from the seven angles. Specifically, the seven angles can be described by a closed-loop trajectory on two different constraint planes under the condition of left-right symmetry (Figure 3). The spatial nature was characterized by the location and orientation of the planes, and the temporal nature by the trajectory on the planes. The location and orientation were determined by the average posture θ_0_ and intersegmental coordination patterns *z*_1_, *z*_2_, and the trajectory by the temporal coordination patterns λ_1_*v*_1_, λ_2_*v*_2_.

The extracted temporal coordination patterns λ_1_*v*_1_, λ_2_*v*_2_ showed no apparent effect of the speed condition irrespective of the phase and gait (Table 3). This implies that the shape of the trajectory remained on the constraint planes. Such temporal invariance has been observed in curved walking when compared with straight-ahead walking (Courtine and Schieppati, 2004). In contrast, the extracted intersegmental coordination patterns *z*_1_, *z*_2_ showed apparent variance with the gait speed in each phase (Table 3). Previous works (Bianchi et al., 1998; Ivanenko et al., 2007, 2008) have shown that the orientation of the constraint plane for three elevation angles of one leg varies for the gait and speed. The orientation of the constraint planes of the seven elevation angles for the whole body also changes for the gait and speed. Furthermore, the DS phase for walking and FL phase for running had larger changes in the plane orientation for speed than did the SS phase [0.97 ± 0.01 (95%CI) and 1.00 ± 0.00 (95%CI) for the DS and SS phases, respectively, of walking and 0.97 ± 0.00 (95%CI) and 0.99 ± 0.00 (95%CI) for the FL and SS phases, respectively, of running] (Table 4). The average posture amplitude |θ_0_| and intersegmental pattern ${\hat{\theta }}_{0}$ also showed apparent speed effects in each phase (Table 5). From a comparison of these characteristics between the DS and SS phases for walking and SS and FL phases for running, the DS phase has larger changes in |θ_0_| and ${\hat{\theta }}_{0}$ than the SS phase for walking [0.25 ± 0.01 (95%CI) and 0.05 ± 0.01 (95%CI) for the DS and SS phases, respectively, in |θ_0_| and 0.98 ± 0.00 (95%CI) and 0.99 ± 0.00 (95%CI) for the DS and SS phases, respectively, in ${\hat{\theta }}_{0}$], similar to the orientation of the constraint planes (Table 6). In contrast, while the SS phase has a larger change in |θ_0_| than the FL phase for running [0.77 ± 0.03 (95%CI) and 0.63 ± 0.01 (95%CI) for the SS and FL phases, respectively], the FL phase has a larger change in ${\hat{\theta }}_{0}$ than the SS phase [0.988 ± 0.001 (95%CI) and 0.992 ± 0.001 (95%CI) for the FL and SS phases, respectively]. These results suggest that, to change speed, humans tune their locomotor kinematics largely in the gait-specific phases.

A comparison of the kinematic coordination patterns in the SS phase between walking and running revealed that while the temporal coordination patterns λ_1_*v*_1_, λ_2_*v*_2_ were similar, the intersegmental coordination patterns *z*_1_, *z*_2_ and the average posture amplitude |θ_0_| and pattern ${\hat{\theta }}_{0}$ differed (Table 7). In particular, the amplitude |θ_0_| had a large difference and was larger for running than for walking [e.g., 0.86 ± 0.01 (95%CI) at 3 km/h for walking and 1.70 ± 0.03 (95%CI) at 9 km/h for running], as shown in Figures 6A,B. This suggests that while walking uses an erect posture, running uses a bent posture. In addition to this difference, the foot of the stance leg and the thigh and shank of the swing leg in *z*_1_ and the thigh and shank of the stance leg in *z*_2_ had clearly different contributions between the gaits (Figure 5). Specifically, the foot had a larger contribution for running in the stance leg of *z*_1_. In the swing leg of *z*_1_, while the shank had a larger contribution than the thigh for walking, the shank and thigh had similar contributions for running. In the stance leg of *z*_2_, while the thigh had a larger contribution for walking, the shank had a larger contribution for running. While the first intersegmental coordination pattern *z*_1_ mainly contributed to the limb axis orientation, the second intersegmental coordination pattern *z*_2_ contributed to the limb axis length. The foot movement of the stance leg in running is larger than that in walking and contributes to the limb axis rotation. For the swing leg, while the thigh movement is larger than the shank movement for walking, they are comparable for running. From $z_{2}{\left(\lambda_{2}v_{2}\right)}^{\text{T}}$, this induced knee extension and then flexion sequentially for walking, which corresponds to the last half of the movement of the double-knee action. In contrast, for running, this induced sequential knee flexion and extension, which corresponds to the spring-like knee bending. These differences reflect different movements between the gaits.

The central pattern generator (CPG) in the spinal cord is largely responsible for adaptive motor control in locomotion (Orlovsky et al., 1999). It has been suggested that the CPG consists of hierarchical networks that include the rhythm generator (RG) and pattern formation (PF) networks (Burke et al., 2001; Lafreniere-Roula and McCrea, 2005; Rybak et al., 2006a,b). The RG network generates the locomotion rhythm in response to sensory feedback, while the PF network shapes the rhythm into spatiotemporal motor patterns through interneurons. The CPG separately controls the spatial and temporal patterns in the RG and PF networks, respectively. In this study, we investigated the adaptation mechanism that produces different speeds in human walking and running by extracting low-dimensional structures from measured kinematics data with singular value decomposition to study the kinematic spatiotemporal coordination patterns. The singular value decomposition divides the data into spatial and temporal patterns on an orthonormal basis, which is useful for elucidating the underlying mechanism for such spatiotemporal patterns. In particular, our results revealed invariant features in the temporal coordination patterns and variant features in the spatial coordination patterns, which show different control strategies for the spatial and temporal patterns in the CPG. In addition to the kinematics data, the analysis of electromyographic data, which reflects motor control strategies more directly than kinematics data, has also shown low-dimensional structures for different walking and running speeds (Cappellini et al., 2006; Hagio et al., 2015; Yokoyama et al., 2016, 2017). These results suggest coordinated motor control patterns and provided useful insights for the adaptation mechanisms in locomotion. Because human locomotion is generated through the control of a redundant musculoskeletal system, the analysis of the low-dimensional coordination structures is useful. In addition to the analysis of measured data, modeling approaches also provided useful insights for the mechanism that forms the low-dimensional structures from a mathematical viewpoint (Jo and Massaquoi, 2007; Barliya et al., 2009; Neptune et al., 2009; Aoi et al., 2010, 2019; Aoi and Funato, 2016). We would like to integrate the measured data analysis and modeling approach to further clarify the adaptation mechanism for locomotion in the future.

## Data Availability

The datasets generated for this study are available on request to the corresponding author.

## Ethics Statement

This study was approved by the Ethics Committee of Doshisha University. Written informed consent was obtained from all participants after the procedures had been fully explained.

## Author Contributions

HO, SA, and KT developed the study design. HO analyzed the data in consultation with SA, TF, NT, and KT. HO and SA wrote the manuscript. All the authors reviewed and approved it.

### Conflict of Interest Statement

The authors declare that the research was conducted in the absence of any commercial or financial relationships that could be construed as a potential conflict of interest.
